# Pluripotent and Metabolic Features of Two Types of Porcine iPSCs Derived from Defined Mouse and Human ES Cell Culture Conditions

**DOI:** 10.1371/journal.pone.0124562

**Published:** 2015-04-20

**Authors:** Wei Zhang, Yangli Pei, Liang Zhong, Bingqiang Wen, Suying Cao, Jianyong Han

**Affiliations:** 1 State Key Laboratory for Agrobiotechnology, College of Biological Sciences, China Agricultural University, Beijing, China; 2 College of Animal Science and Technology, Beijing University of Agriculture, Beijing, China; National University of Singapore, SINGAPORE

## Abstract

The domestic pig is an excellent animal model for stem cell research and clinical medicine. There is still no suitable culture condition to generate authentic porcine embryonic stem cells (pESCs) and high quality porcine induced pluripotent stem cells (piPSCs). In this study, we found that culture conditions affected pluripotent and metabolic features of piPSCs. Using defined human embryonic stem cell (hESC) and mouse ESC (mESC) culture conditions, we generated two types of piPSCs, one of which was morphologically similar to hESCs (here called hpiPSCs), the other resembled mESCs (here called mpiPSCs). Transcriptome analysis and signaling pathway inhibition results suggested that mpiPSCs shared more of mESC signaling pathways, such as the BMP pathway and JAK/STAT pathway and hpiPSCs shared more hESC signaling pathways, such as the FGF pathway. Importantly, the mpiPSCs performed embryonic chimera incorporation more efficiently than the hpiPSCs did. In addition, the mpiPSCs showed mitochondrial features of naive ESCs and lipid droplets accumulation. These evidences may facilitate understanding of the gene regulation network and metabolism in piPSCs and promote derivation of *bona fide* pESCs for translational medicine.

## Introduction

Naïve and primed states are the two states of pluripotent stem cells. The naïve mouse embryonic stem cells (mESCs) derived from early embryo are significantly different from primed human ESCs (hESCs) and mouse epiblast stem cells (EpiSCs) in morphology, patterns of gene expression and metabolism[1]. The leukemia inhibitory factor (LIF) is necessary for mESCs pluripotency maintenance [2–4]. Sustaining the undifferentation state of hESCs depends on basic FGF (bFGF) [5, 6]. However, rat ES cells have been derived from N2B27 medium containing either 3i (FGF receptor inhibitor SU5402, MEK inhibitor PD184352 and GSK3 inhibitor CHIR99021) plus LIF or 2i (PD0325901 and CHIR99021) plus LIF [7]. Recent reports have shown that naïve hESCs can be derived from embryo or converted from primed hESCs using defined culture medium containing a series of small molecules [8, 9]. These findings have demonstrated that specific culture conditions are necessary for maintenance the pluripotent state of hESCs and mESCs.

Many efforts have been made to derive authentic pig ESCs, but no conclusive results have been produced so far. When iPSCs technology was created, piPSCs were expected to provide an alternative resource of pESCs to advance regenerative medicine research from the bench to clinical use [10]. Ezashi et al. first derived bFGF-depended piPSCs and their physiology was similar to hESCs [11]. The mESC-like piPSCs can be produced in 2i plus LIF medium [12]. However, the exact difference between the two types of piPSCs with respect to pluripotent and metabolic features had not yet been determined. In the current study, we generated two types of porcine iPSCs using hESC and mESC culture conditions respectively. The two types of piPSCs showed different gene expression patterns and depended on different signaling pathways for maintaining stem cell state. More importantly, mitochondrial features and lipid droplets accumulation differed in the two types of piPSCs, which indicated that they had different metabolic features. These results suggested that the culture conditions are one determinant of the pluripotent state of piPSCs.

## Materials and Methods

### Animals

Young adult female Nong Da Xiang pigs (China Agricultural University pig farm, Zhuo Zhou, China) and adult female CF1 mice (Vital River Laboratories, Beijing, China) were used to produce the embryonic fibroblasts. All animal experiments in the present study were approved by the Animal Care and Use Committee of China Agricultural University.

### Cell culture

The porcine embryonic fibroblasts (pEFs) were derived from day 26–30 embryos using a standard procedure, maintained in DMEM medium containing 10% fetal bovine serum (FBS), 1% non-essential amino acid, 1% GlutaMAX-L and 1% penicillin/streptomycin (Gibico)[13]. The piPSCs generated in this study were cultured in hESC medium (containing bFGF) or mESC medium (2i plus LIF). The hESC medium was the commercial defined medium (NutriStem XF/FF medium, Stemgent 01–0005), mESC medium contained N2B27 (Gibico), 5 ng/ml human LIF (Millipore, LIF1005) and 3 μM CHIR99021 (Selleck, S1036), 2 μM PD0325901 (Selleck, 252917). The hpiPSCs were passaged by 0.1% collagenase Ⅳ(Gibico) and mpiPSCs by TrypLE (Gibico). Both types of piPSCs were cultured on feeder cells. The 293-GP2 cells used for viral packaging were cultured in DMEM with 10% FBS.

### Retrovirus production and piPSCs generation

Retroviral virus vectors (pMXs system) separately carrying porcine *Oct4*, *Sox2*, *Klf4* and *Myc* were here used to reprogram pEFs. Viral production was performed using a method described previously [13]. Retroviruses were used to infect pEFs for 12 h in presence of 8 μg/ml polybrene (Sigma, 107689). After two rounds of infection, infected cells were passaged and seeded into mitomycin C inactivated mouse embryonic fibroblast-coated plates, followed by changing to mESC medium or hESC medium on the second day. The medium was changed every two days. The reprogramming efficiency was evaluated by AP staining at day 15. Colonies were picked up at day 30 (S1A Fig).

### Alkaline Phosphatase staining and Karyotype analysis

The alkaline phosphatase (AP) activity of piPSCs was determined using an Alkaline Phosphatase Detection Kit (Millipore, SCR004) according to the manufacturer’s instructions. For karyotype analysis, the cells were treated with KaryoMAX Colcemid Solution (Gibico) for 3 h, then were collected and incubated in 0.075 mol/L KCl for 20 min at 37°C. After centrifugation at 1500 r/min for 5 min, cell pellets were fixed in cold 1:3 (glacial acetic acid: methanol) solution for 15 min and this process was repeated three times. For metaphase analysis, fixed cells were dropped on cold slides, and dried at room temperature. The slides were stoving at 75°C for 3 h then treated with 0.025% Trypsin. Finally, the samples were stained with 10% Giemsa.

### Immunocytochemistry

For immunocytochemical analysis, cells were fixed with 4% paraformaldehyde (PFA) in DPBS for 20 min at room temperature. Fixed cells were washed three times with DPBS, incubated in 0.2% Triton X-100 buffer for 15 min, and washed three times. After blocked in 2% BSA blocking buffer for 30 min, cells were incubated in 1% BSA buffer containing primary antibodies at 4°C overnight. The following primary antibodies were used: anti-Oct4 (Santa cruz, sc-5297), anti-Sox2 (Abcam, ab97959), anti-SSEA-1 (Cell Signaling Technology, 4744S), anti-SSEA-4 (Cell Signaling Technology, 4755P), anti-Tra-1-81 (Cell Signaling Technology, 4745P), anti-Tra-1-60 (Cell Signaling Technology, 4746P) and anti-H3K27me3 (Abcam, ab6002). The cells were washed twice in DPBS and stained for 1 h in secondary antibody, Alexa Fluor 488 (or 594) Goat Anti-Mouse IgG (H+L) or Alexa Fluor 488 (or 594) Goat Anti-rabbit IgG (H+L) (Life Technologies). For nuclear staining, the cells were incubated for 2 min with Hoechst 333342 (10 ng/ml) (Life Technologies, 1046265). The images were captured by Nikon A1 microscope.

### Quantitative RT-PCR analysis

Total cellular RNAs of porcine cells were extracted using an RNeasy Mini Kit (QIAGEN, 74104). The reverse transcription was performed using Oligo-dT primer and M-MLV Reverse Transcriptase (Promega, M1701). Quantitative RT-PCR analysis were performed using LightCycler 480 SYBR Green I Master Kit (Roche, 4887352001), and detected with Light cycler 480Ⅱ(Roche). The data was analyzed using the comparative CT (2^-ΔΔCT^) method. The ΔCT was calculated using EF1-α as internal control. All experiments were performed more than three biological replicates. Primer sequences were provided in S1 Table.

### Embryoid body and teratoma formation assay

The piPSCs were digested into single cell suspension, seeded into low-adhesive 10 cm plate and cultured in embryoid body forming medium consisting of DMEM with 10% FBS, 1% NEAA, 1% GlutaMAX-L and 1% penicillin/streptomycin (Gibico). Low-adhesive culture plates were placed on a shaker (40 r/min) in a CO_2_ incubator. After two days, the embryoid bodies were seeded into 1% gelatin-coated plate and cultured in EB forming medium for 10 more days, then fixed for immunocytochemistry. Antibody anti-Neuron specific β III Tubulin antibody (Abcam, ab18207), anti-α-Smooth muscle actin (SMA) (Abcam, ab5694) and anti-Sox17 (Millipore, 09–038) were used for detection of 3 germ-layer-cells. For teratoma formation, the piPSCs (2×10^7^) were subcutaneously injected into the BALB/c nude mice. After two months, the mice were sacrificed by carbon dioxide (CO_2_) inhalation. The teratoma were dissected and fixed in 4% PFA. The fixed samples were embedded in paraffin and sections were hematoxylin and eosin staining.

### Parthenogenetic embryo injection

The maturation and parthenogenetic activation (PA) of porcine oocytes were performed as described previously [14]. After 3 days of PA, 8-cell stage embryos were selected for injection assay. About 15–20 piPSCs were injected into each embryo by micromanipulation. The injected embryos were then transferred into PZM-3 medium for 3–4 days. The number of blastocysts and chimeric blastocysts was counted and analyzed.

### Transcriptome analysis

The transcriptional profiles of hpiPSCs, mpiPSCs and pEFs were analyzed to evaluate their pluripotent state. Total RNA were extracted by RNeasy Mini Kit (QIAGEN, 74104). Affymetrix GeneChip Porcine Genome Arrays were used, and all experiments were performed at Beijing Capitalbio Corporation. The RMA method from R package: *affy* was used for data normalization and background corrected expression values calculation [15]. The *limma* (Linear Models for Microarray Data) package from Bioconductor was used to analyze the differential gene expression [16]. Annotation and enrichment were analyzed using the *GOstats* package [17]. Differential genes associated with specific pathways based on the Kyoto Encyclopedia of Genes and Genomes (KEGG) were also analyzed.

### Ultrastructural analysis by electron microscope

The piPSCs were fixed with 2.5% glutaraldehyde for 3 h, embedded in 4% agar at 45°C, and post-fixed in 1% OsO_4_ for another 3h. The embedded cells were then stained with 1% uranyl acetate and dehydrated serially in ethanol. The cells were washed twice in propylene oxide for 10 min, further embedded in Epon and polymerized for 48 h at 60°C for sectioning. Ultrathin sections were stained with 2% uranyl acetate and lead citrate, and then observed under JEM-100CX transmission electron microscope and photographed.

### Mitochondria and lipid droplet distribution analysis

The two types of piPSCs were digested, seeded 3×10^3^ cells per well into 96-well plate and detected at day 3. The mitochondria were stained by Mitotracker Deep Red FM (Life Technologies, M22426) according to the manufacturer’s instructions. The lipid droplets were stained using Nile Red (Sigma, N-1142). The cells were fixed with 4% PFA for 20 min at room temperature. Cells were washed three times with DPBS and then incubated with Nile Red solution (5 ng/ml) for 5 min at room temperature. To quantify the accumulation of cytoplasmic Nile Red and Mitotracker fluorescence in acquired images, the high content screening system “Operetta” was used in combination with the integrated image analysis software “Harmony” of Perkin–Elmer. From these images the intensity of Nile Red and Mitotracker fluorescence in the cytoplasmic area of each cell could be quantified by the software.

### Signaling pathway identification experiment

The cells were incubated with 10 μM FGFR inhibitor AZD4547 (Selleck, S2801), 5 μM BMP signaling pathway inhibitor Dorsomorphin (Selleck, P5499) or 10 μM JAK inhibitor Ruxolitinib (Selleck, S1378) for 3 days, then passaged once. The same number of cells (1×10^4^ cells per well) was seeded into 6-well plate. Inhibitors were added to the cells and allowed to incubate for 3 days. AP staining was performed and pictures were captured. DMSO was a negative control group. Medium was changed daily

### Statistical analysis

Values were presented as the mean ± SD. Statistical significance was assessed by using Student’s test where indicated in the figure legends.

## Results

### The mpiPSCs and hpiPSCs present different ES cell features

**Fig 1:**
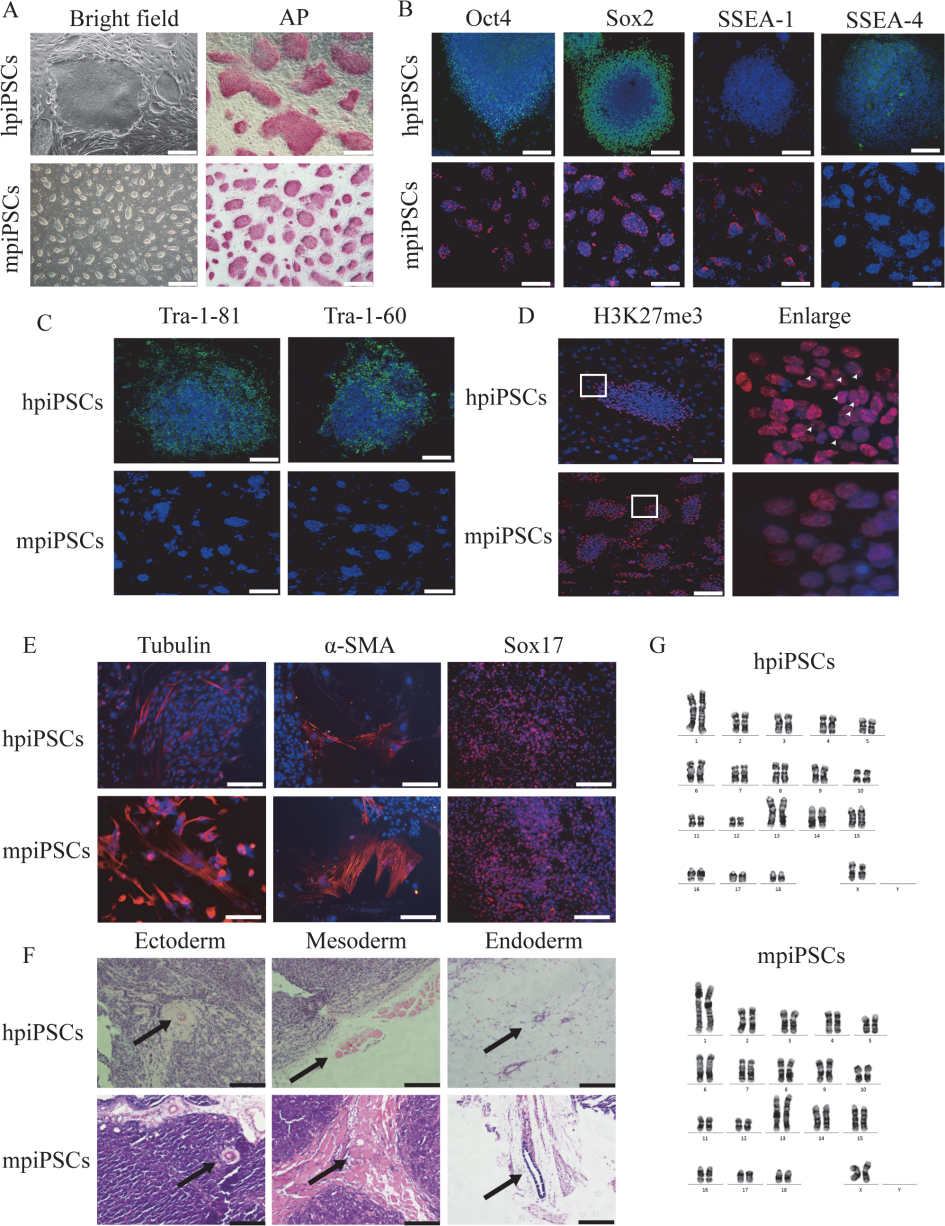
Characterization of the two types of piPSCs. (A)The colonies of mpiPSCs were round and three-dimensional (bottom). The colonies of hpiPSCs were large and flat (top). Bar scale = 500 μm. The hpiPSCs and mpiPSCs were both positive for alkaline phosphatase (AP). Bar scale = 200 μm. (B) Immunocytochemical staining of Oct4, Sox2, SSEA-1 and SSEA-4 in hpiPSCs and mpiPSCs. Bar scale = 200 μm. (C) The surface marker Tra-1-81 and Tra-1-60 were positive in hpiPSCs but not detected in mpiPSCs. Bar scale = 200 μm. (D) X chromosome activation state of hpiPSCs and mpiPSCs after immunostaining for H3K27me3. Positive signals of histone H3K27 me3 spots were observed in hpiPSCs but not in mpiPSCs. Bar scale = 200 μm. The white arrowheads indicate the H3K27me3 positive spot in the cell. (E) Immunocytochemical assay of 3-germ-layer cells in EBs derived from both types of piPSCs, the markers include β Ⅲ-Tubulin (ectoderm), α-SMA (mesoderm) and Sox17 (endoderm). Scale bar = 200 μm. (F) Hematoxylin and eosin staining of teratoma sections of piPSCs. Left: blood vessel of endothelium (ectoderm); middle: muscle (mesoderm); right: gut-like epithelium (endoderm). Scale bars = 200 μm. (G) Karyotype analysis of piPSCs.

The hpiPSCs cultured in hESC medium were found hESC-like or epiblast-type morphology and the colonies were large and flat. However, the mpiPSCs cultured in mESC medium were much more similar to mESC-like morphology. They were three-dimensional and rounded (Fig 1A: left). The hpiPSCs usually were digested into small clumps by collagenase for passage. The mpiPSCs could be digested to single cell with TrypLe or 0.25% trypsin. Both hpiPSCs and mpiPSCs were alkaline phosphatase (AP) staining positive (Fig 1A: right), and were able to be maintained *in vitro* for more than 30 passages. The hpiPSCs expressed hESC pluripotent markers, including stage-specific embryonic antigen SSEA-4, transcription factors Oct4 and Sox2, but hpiPSCs did not express mESC cell-specific surface antigen SSEA-1(Fig 1B: top). The mpiPSCs exhibited SSEA-1 positive as well as Oct4 and Sox2, whereas SSEA-4 was negative (Fig 1B: buttom). The hpiPSCs highly expressed hESC membrane markers Tra-1-81 and Tra-1-60 but they were negative in mpiPSCs (Fig 1C). The activation of X chromosome in cells was estimated using immunofluorescent staining of H3K27me3 spots in nucleus. The fluorescence intensity of H3K27me3 spots was observed in hpiPSCs, but it wasn’t observed in mpiPSCs (Fig 1D). These results suggested that mpiPSCs were similar to the naïve state than hpiPSCs. Differentiation experiment *in vitro* showed both kinds of piPSCs were able to form embryoid bodies and differentiate into 3-germ-layer cells (Fig 1E). Both kinds of piPSCs developed into teratomas in immunodeficient mice. Hematoxylin and eosin staining assay suggested that teratomas contained 3-germ-layer cells (Fig 1F). Both the mpiPSCs and the hpiPSCs showed a normal karyotype (Fig 1G).

### The mpiPSCs performed embryonic chimera incorporation more efficiently than the hpiPSCs did

**Fig 2:**
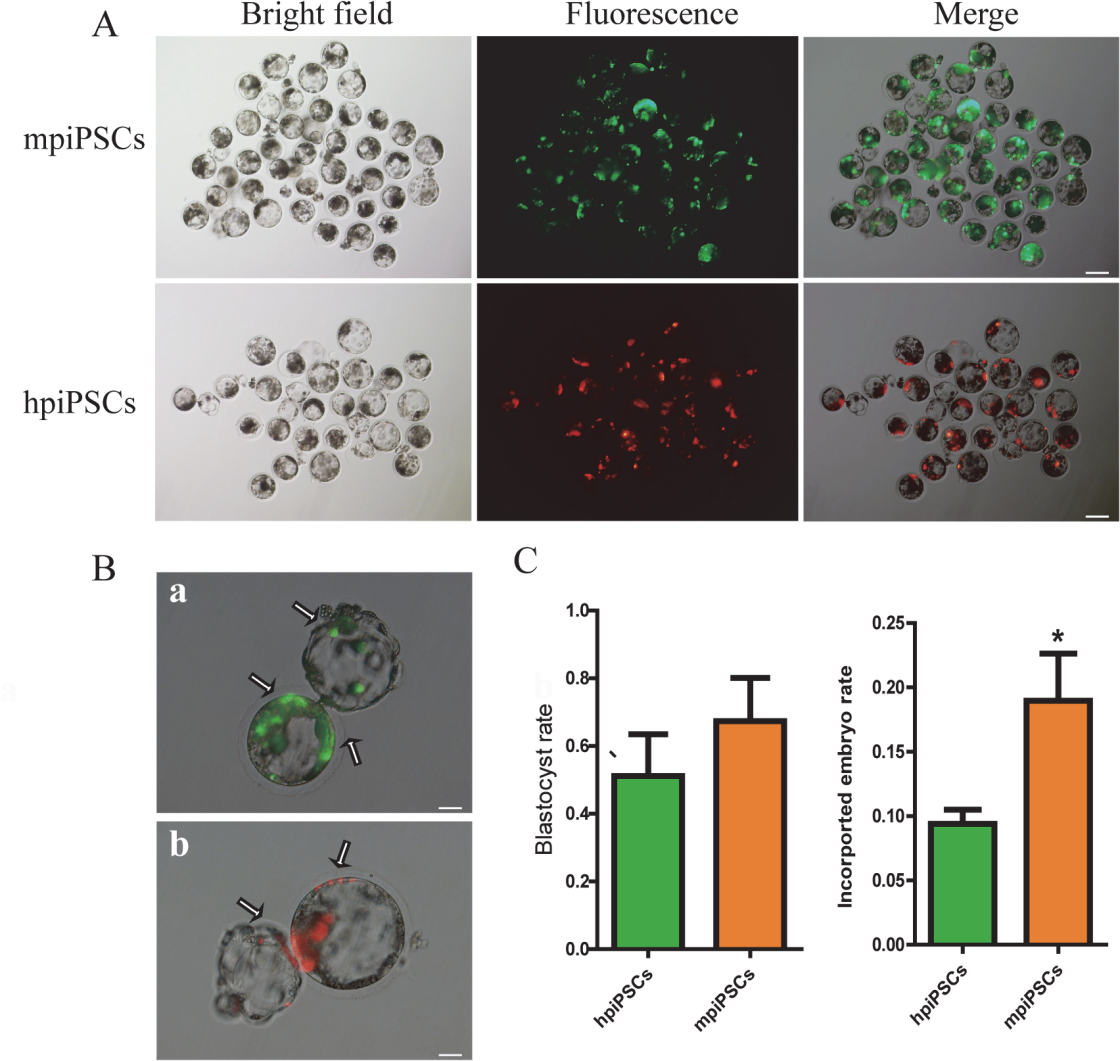
Injection of piPSCs into parthenogenetic embryos and their development *in vitro*. (A) The mpiPSCs were injected into porcine PA embryos. The mpiPSCs were EGFP-positive in the PA blastocysts (top). The hpiPSCs were CM-Dil (Red)-positive in the PA blastocysts (bottom). Scale bar = 100 μm. (B) The two types of piPSCs were incorporated in the PA embryo in vitro. (a): The mpiPSCs (EGFP) were incorporated into the blastocyst, in both ICM and trophoblast (TE) (arrows indicated). (b): The hpiPSCs (Red) were incorporated into the blastocyst (arrows indicated). Scale bars = 50 μm. (C) The blastocyst rate (blastocyst numbers / total injected embryo numbers) was no difference between the two types of piPSCs. The incorporated embryo rate (the incorporated embryo numbers / total injected embryo numbers) of mpiPSCs was significantly higher than hpiPSCs. * *p* < 0.05, (Mean ± SD, n = 3).

The mpiPSCs were labeled with EGFP fluorescence protein and the hpiPSCs were stained with cell tracker CM-Dil. They were then injected into porcine parthenogentic embryo at 8-cell stage to exam their ability of contribution to the inner cell mass (ICM) and trophectoderm (TE) when the embryos developed into blastocysts (Fig 2A). Two types of piPSCs were able to incorporate into ICM and TE of hatching blastocysts (Fig 2B). The developed blastocyst rate had no difference between mpiPSCs and hpiPSCs. However, mpiPSCs had a higher rate of incorporation than hpiPSCs did (Fig 2C). This demonstrated that mpiPSCs may be more suitable as donor cells for chimera manipulation than hpiPSCs *in vitro*. However, we didn’t get any chimeric piglets generated with both of the piPSCs. This may be due to continue expression of the transgenes, which was a main barrier to generate naïve piPSCs though low-chimeric piglets were produced with bFGF-depended piPSCs [18].

### Transcriptome reveals that mpiPSCs were more similar to porcine early ICM than hpiPSCs

**Fig 3:**
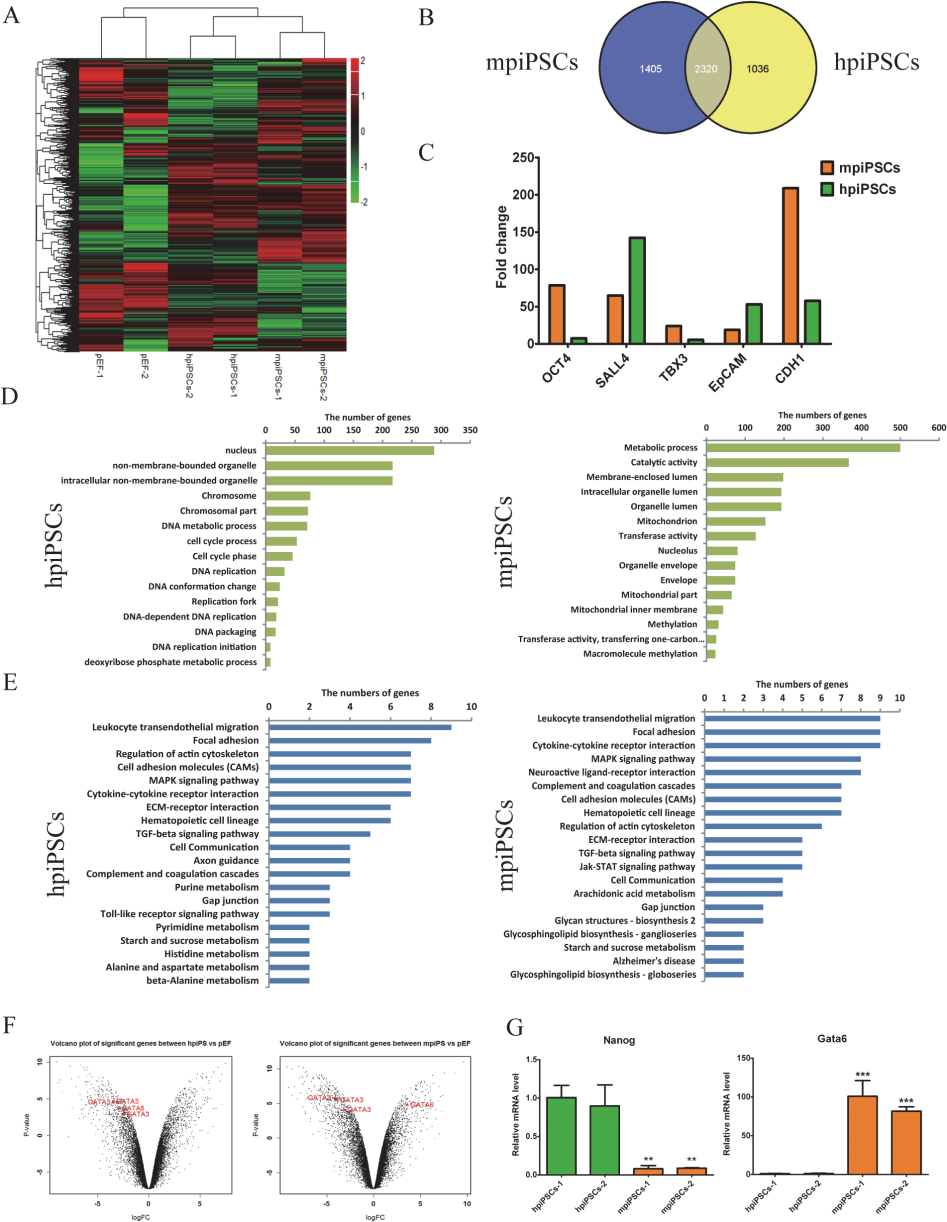
Gene expression profiling of hpiPSCs and mpiPSCs. (A) Heatmap of differential genes of mpiPSCs, hpiPSCs and pEFs. (B) Venn diagram showed gene expression pattern in mpiPSCs and hpiPSCs more different than that in pEFs. (C) The typical pluripotent genes such as *Oct4*, *Sall4*, *Tbx3*, *Epcam* and *CDH1* were up-regulated in both types of piPSCs compared with pEFs (fold change > 2). (D) GO term analysis of piPSCs versus pEFs (p value <0.01). (E) KEGG pathway analysis of differential genes from piPSCs versus pEFs (p value < 0.01). (F) Expression levels of *Gata3* and *Gata6* in hpiPSCs and mpiPSCs. *Gata3* and *Gata6* were low in hpiPSCs (Log_2_ fold change value < -1). The level of expression of *Gata3* was low in mpiPSCs (Log_2_ fold change value < -1), but the expression of *Gata6* was higher than *Gata3* in mpiPSCs (Log_2_ fold change value > 1). (G) Relative expression levels of *Nanog* and *Gata6* in mpiPSCs and hpiPSCs were evaluated by quantitative RT-PCR assay. The mRNA levels were normalized to EF-1α. Relative mRNA level of *Nanog* in hpiPSCs was more significantly up-regulated in hpiPSCs than that in mpiPSCs. Relative mRNA level of *Gata6* was down-regulated in hpiPSCs. ***p < 0.001 (mean ± SD, n = 3).

To further investigate the molecular mechanisms underlying two types of piPSCs, we performed microarray analysis and compared their global gene expression profiles. The results demonstrated that both piPSCs cell lines clustered differently from somatic cell lines. Heatmap indicated obvious difference between hpiPSCs and mpiPSCs (Fig 3A and S3 Fig). As compared with pEFs, 1405 differential genes were specific expressed in mpiPSCs and 1036 differential genes in hpiPSCs. 2320 common genes were expressed in both types of piPSCs (Fig 3B). The pluripotent genes such as *Oct4*, *sall4*, *Tbx3*, *Epcam* and *CDH1* (*E-cadherin*) were up-regulated in both piPSCs (fold change > 2) (Fig 3C). GO analysis of differential genes (fold change > 2) in piPSCs versus pEFs was categorized in Fig 3D. The most enriched genes are relevant with mitochondrion, methylation and nucleolus in mpiPSCs. Genes involved in DNA replication and DNA metabolic process were enriched in hpiPSCs. KEGG pathway analysis was performed for the differential genes from two types of piPSCs versus pEFs. In top 20 categories (*p* value < 0.01) there were significantly more genes involved in JAK/STAT signaling pathway, arachidonic acid metabolism and glycosphingolipid biosynthesis in mpiPSCs. Genes relevant with Toll-like receptor signaling pathway, axon guidance, purine metabolism and beta-Alanine metabolism were enriched in hpiPSCs (Fig 3E). Results also showed that *Gata6*, a primitive endoderm (PE) marker, was up-regulated in mpiPSCs but not in hpiPSCs. Meanwhile, *Gata3* showed low expression level in both types of piPSCs (Fig 3F). Quantitative RT-PCR assay confirmed that the mRNA level of *Gata6* was much higher in mpiPSCs than in hpiPSCs (Fig 3G: right). In the early human and mouse embryos, *Nanog* and *Gata6* showed distinct expression patterns. *Gata6*-positive cells in ICM showed low level expression of *Nanog* [19]. However, Chan et al. described that naïve hESCs derived from defined culture condition showed obvious co-expression of *Nanog* and *Gata6* [20].There was no probe for *Nanog* in the microarray chip, so endogenous *Nanog* was detected through the quantitative RT-PCR assay. The results indicated that the mRNA level of *Nanog* in hpiPSCs was much higher than in mpiPSCs (Fig 3G: left). This was consistent with previous reports that the expression level of *Nanog* was much higher in primed PSCs than that in the putative naïve or m piPSCs [21, 22]. In porcine embryos, *Nanog* showed less expression in ICM but higher expression in epiblasts [23]. These results indicated that mpiPSCs was much more similar to the porcine ICM than hpiPSCs. Expression levels of four endogenous genes, *Oct4*, *Sox2*, *Klf4* and *Myc*, had also been detected. Endogenous *Oct4*, *Sox2* and *Myc* were up-regulated but *Klf4* didn’t be activated in both types of piPSCs (S2A Fig). However, the transgenes of piPSCs were not silenced in two kinds of piPSCs as mouse iPSCs did (S2B Fig).

### The mpiPSCs depend on the JAK/STAT and BMP signaling pathways while hpiPSCs depend on the FGF signaling pathway

**Fig 4:**
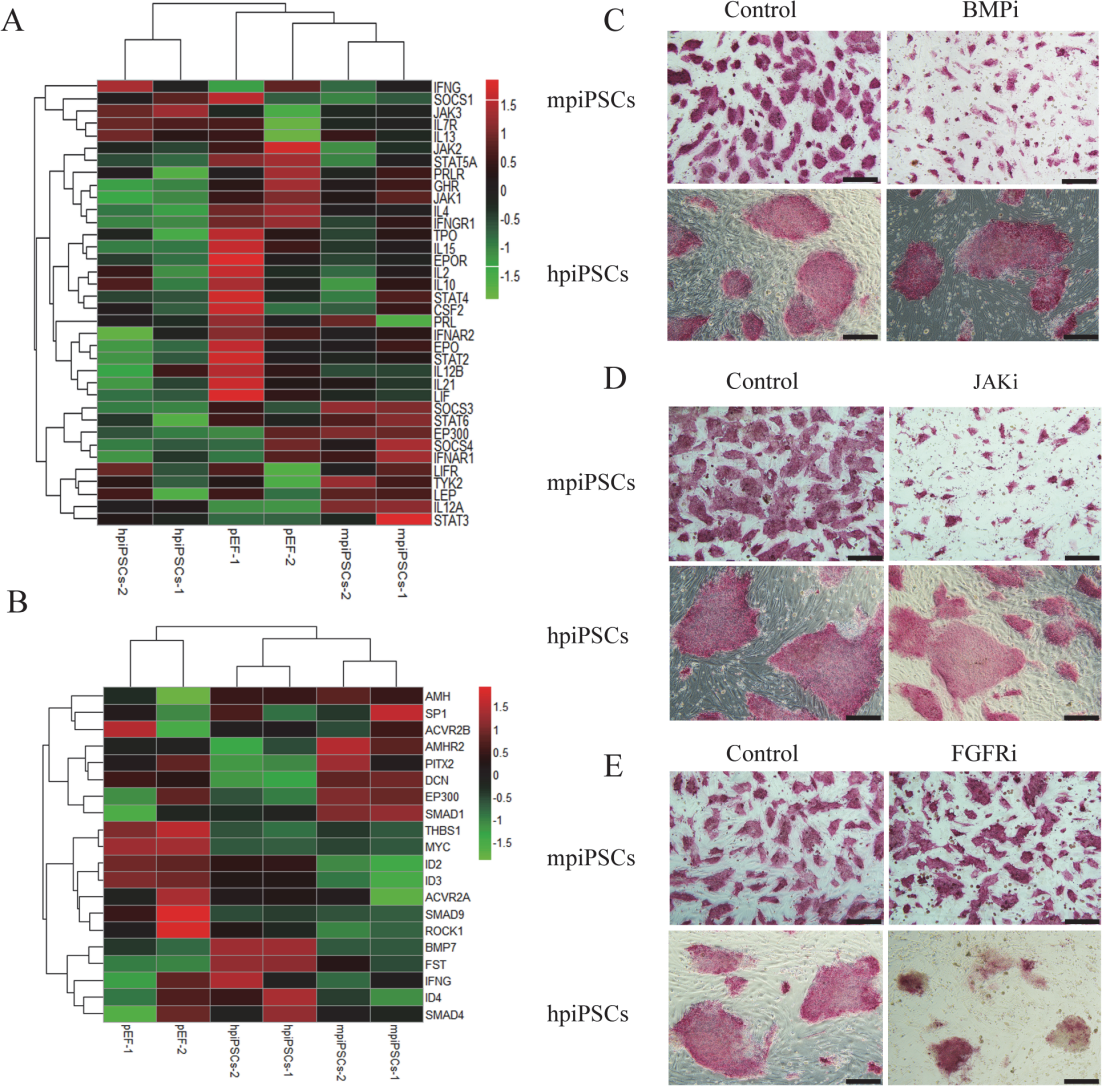
The mpiPSCs and hpiPSCs depended on different signaling pathways. (A) Heatmap of selected genes involved in the JAK/STAT signaling pathway. (B) Heatmap of selected genes involved in FGF/BMP/TGF-beta signaling pathways in mpiPSCs, hpiPSCs and pEFs. (C) AP staining for mpiPSCs and hpiPSCs in BMP inhibitor and control group. Bar scale = 200 μm. (D) AP staining for piPSCs in JAK inhibitor and control group. Bar scale = 200 μm. (E) AP staining for piPSCs cultured in FGFR inhibitor and control group. Bar scale = 200 μm.

It is reported that mESCs depended on the JAK/STAT pathway and BMP pathway to keep self-renewal and pluripotency. BMP4 was able to coordinate with LIF to maintain mESC pluripotency in a serum-free culture condition [24–26]. Additionally, hESCs were found to depend on FGF signaling pathway [27–29]. According to bioinformatics analysis, the LIFR (LIF Receptor), *Stat3* and other genes involved in JAK/STAT signaling pathway were up-regulated in mpiPSCs (Fig 4A). Up-regulation of *Smad1* in mpiPSCs suggested that the BMP signaling pathway might be activated (Fig 4B). The effect of FGFR inhibitor (AZD4547), BMP signaling pathway inhibitor (Dorsomorphin) and JAK inhibitor (Ruxolitinib) on the two types of cells was tested. When the Dorsomorphin and Ruxolitinib were added to mpiPSCs culture medium, the morphology of mpiPSCs changed obviously. The colonies became very small and abnormal, but the hpiPSCs did not show any changes (Fig 4C and 4D). The hpiPSCs could not keep any normal ES-like or AP positive colony in presence of AZD4547 while mpiPSCs grew well within the FGFR inhibitor (Fig 4E). These suggested FGF signaling pathway was indispensable to hpiPSCs.

### Mitochondrial remodeling and lipid droplets accumulation were observed in mpiPSCs

**Fig 5:**
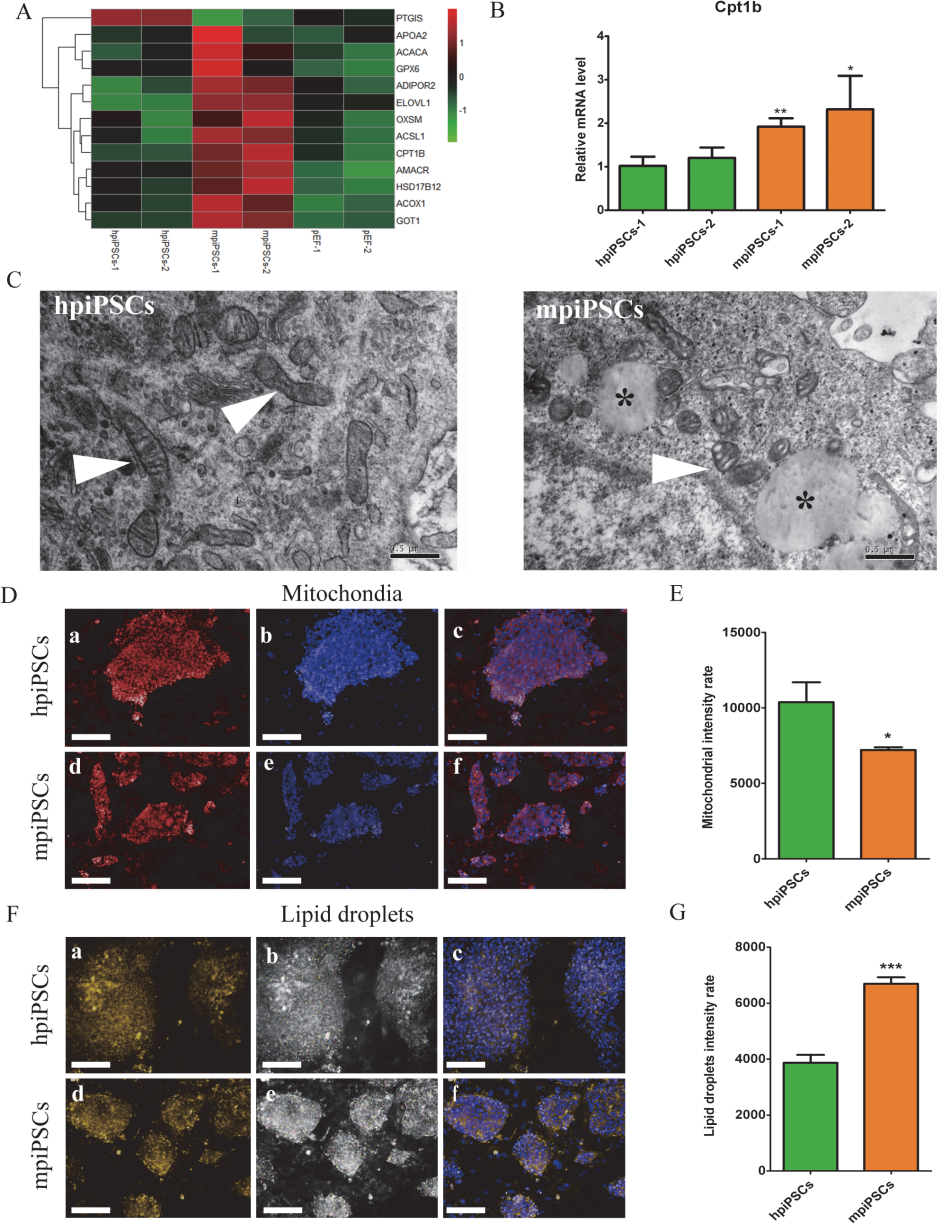
Metabolic features of the two types of piPSCs. (A)Heatmap of selected genes involved in metabolism. (B) Relative mRNA levels of *Cpt1b* in hpiPSCs and mpiPSCs. *p < 0.05, **p < 0.01 (mean ± SD, n = 3) (C) Electron microscope images of hpiPSCs and mpiPSCs. White arrowheads indicate for mitochondria and black asterisks indicate lipid droplets. Bar scale = 0.5 μm. (D) The Mitotracker staining of piPSCs. Mitotracker deep Red (a and d), Hoechst (b and e), overlay (c and f). Bar scale = 100 μm. (E) Mitochondrial membrane intensity rate (intensity of fluorescence / cell areas (μm^2^)) of piPSCs. *p < 0.05 (mean ± SD, n = 3) (F) The Nile Red staining of piPSCs. Nile Red (a and d), lipid droplets detected in piPSCs’ colonies by “Harmony” software. Lipid droplets labeled by color circles (b and e), Nile red and Hoechst overlay (c and f). Bar scale = 100 μm. (G) Lipid droplet intensity rate (intensity of fluorescence / cell areas (μm^2^)) of piPSCs. ***p < 0.001 (mean ± SD, n = 3).

Heatmap of differential genes involved in metabolism showed that fatty acid metabolism in mpiPSCs was stronger than that in hpiPSCs. The differential genes related to the fatty acid metabolism, such as *Cpt1b*, were up-regulated in mpiPSCs (Fig 5A). The mRNA levels of *Cpt1b* in mpiPSCs were much higher than that in hpiPSCs and this was confirmed by quantitative RT-PCR (Fig 5B). *Cpt1b* is required for the transport of long-chain fatty acyl-CoAs from the cytoplasm into the mitochondria. The former GO analysis showed that the differential genes were enriched in mitochondria biological process in mpiPSCs. So we checked the morphology of mitochondria in piPSCs. Ultrastructural observation showed that mpiPSCs contained immature mitochondria with few cristae. Mitochondria in hpiPSCs were claviform with more cristae (Fig 5C). Mitotracker Deep Red generally stained on mitochondrial membrane (Fig 5D). The intensity of Mitotracker Deep Red per cell area (μm^2^) was significantly higher in hpiPSCs than in mpiPSCs (Fig 5E). These indicated that the mitochondria have more membrane structures in hpiPSCs than in mpiPSCs. Interestingly, many lipid droplets accumulation were found in cytoplasm of mpiPSCs (Fig 5F). The intensity of Nile Red per mpiPSC was significantly higher than that per hpiPSC (Fig 5G). Lipid droplets accumulated in porcine oocytes and early embryos [30]. We also found that fatty acid metabolism is more active in porcine early embryos development than in human or mouse embryos [31]. Lipid metabolism may play a very important role in porcine pluripotent stem cell proliferation and preimplantation development.

## Discussion

The naïve state of mESCs and primed state of hESCs and mEpiSCs depend on respective suitable culture condition, which was supplemented with growth factors and small molecules that activate special signaling pathways for maintaining self-renewal and pluripotency. No authentic porcine ESCs have yet been established and no piPSCs have been kept stable in culture in the absence of transgenes, this indicated the current culture conditions needed to be further improved. Here, the two sets of traditional culture conditions of mESCs and hESCs used for generation piPSCs were compared. The piPSCs derived from two culture conditions showed different characteristics such as pluripotent markers, the ability of contribution into early embryos, gene expression patterns and metabolic features.

The expression of stem cell surface markers was different between naïve state and primed state, for example, the mESCs expressed SSEA-1 but hESCs expressed SSEA-4, SSEA-3, Tra-1-60 and Tra-1-81 specially [32, 33]. Expression level of SSEA-1 is high in complete ICM and weak in the trophectderm-covered epiblast (EPI) [34]. SSEA-4, Tra-1-60 and Tra-1-81 were not detectable in the porcine ICM or EPI [23]. In this study, SSEA-1 was expressed in mpiPSCs but not in hpiPSCs. This suggested that mpiPSCs was more similar to the porcine ICM. *Xist* gene less expressed in naïve pluripotent stem cells than in primed pluripotent stem cells [1]. The expression of *Xist* gene can induced X chromosome inactivation (XCl) and the region of XCl is accumulated by H3K27 methylation [35]. The fluorescence intensity of H3K27me3 loci in mpiPSCs was much weaker than in hpiPSCs, this means that the mpiPSCs may have two active X chromosomes and that hpiPSCs still have one X chromosome inactivation. This suggested that the pluripotent state of mpiPSCs may be similar to naïve state.

The results of piPSCs incorporation into the ICM of pig PA embryo showed that the efficiency of mpiPSCs was higher than hpiPSCs. Similar results have previously been reported [22]. Another previous work showed that mESCs-like piPSCs have better developmental potential *in vitro* and *in vivo* than the flattened ones, as indicated by testing the efficiency of electroporation transgenes into piPSCs [36]. It indicated that LIF and 2i were necessary for generation of naïve pig pluripotent stem cells, and some more key regulation factors also need to be explored.

In addition, we found that the Gata6 was more highly expressed in mpiPSCs than that in hpiPSCs. Gata family such as *Gata3* and *Gata6* could replace *Oct4* in the reprogramming of mouse and human iPSCs [37, 38]. In early porcine embryos, *Gata6* was expressed only in the porcine ICM [21]. This suggested that *Gata6* may be a marker of pig ICM or pig naive pluripotent stem cells. The present work also showed that the JAK/STAT, FGF and BMP signaling pathways can regulate pluripotency in piPSCs. The mpiPSCs depended on the JAK/STAT and BMP pathways, which is similar with the previous report [39, 40]. Recent report also showed that combination of LIF, BMP4 and two inhibitors (CHIR99021 and SB431542) not only promoted the mesenchymal-to-epithelial transition (MET) during the reprogramming of porcine fibroblasts but also stabilized an intermediate pluripotent state of piPSCs [41]. However, the hpiPSCs require the FGF pathway for maintaining pluripotency. FGF signaling pathway was also critical for pluripotency maintenance and cell proliferation of porcine iPSCs and EpiSCs-like porcine ESCs from blastocyst-stage porcine embryos [23, 42].

Mouse ESCs make use of oxidative phosphorylation but EpiSCs and human ESCs are mostly depend on glycolysis with low mitochondrial respiration capacity. However, the mitochondria of hESCs show more mature in appearance than those of mESCs [43]. When primed hESCs were transformed to the naïve state, the mitochondria appeared less mature and generally rounded with few cristae[9]. Lipid droplets play an important role in embryo development. Lipid droplets store maternal histone protein to protect early embryonic development in *Drosophila* [44, 45]. In the Day 6 porcine ICM, the cytoplasm of the cell was featured by the presence of small rounded mitochondria with few cristae and abundant lipid droplets of varying sizes [34]. This indicated that the cytoplasm of mpiPSCs was similar to that of porcine ICM. However, in the porcine epiblast cells, the lipid droplets were barely detectable in the cytoplasm and the mitochondria were much more numerous and rounded and they presented numerous tubular enfolding of the inner membrane [34]. Similarly, the mitochondria of hpiPSCs also appeared mature feature and showed considerable numerous membrane structures. This reveals that mpiPSCs and hpiPSCs may have metabolic differences. In porcine early embryo, the genes involved in fatty acid biosynthesis and fatty acid metabolism were up-regulated, which was distinct from human and mouse embryos [31]. Similarly, the genes which were relevant with fatty acid metabolism were also activated in the mpiPSCs. It indicated that fatty acid metabolism activation is a marked feature of porcine embryo and pluripotent stem cells.

**Fig 6:**
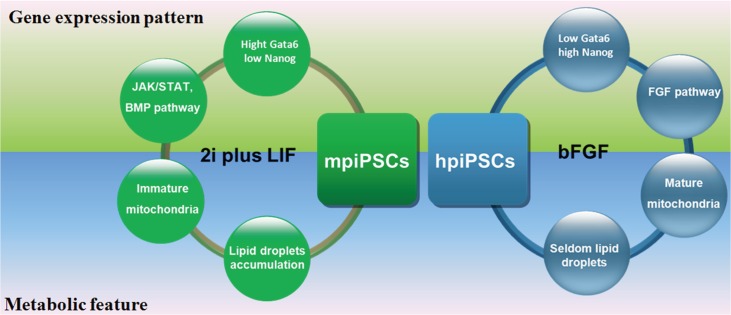
A model showed the difference between two types of piPSCs. The two types of piPSCs derived from mESC medium (2i plus LIF) and hESC medium (containing bFGF) showed the different characteristics in gene expression pattern and metabolic feature.

The two types of piPSCs derived from the mESC medium and hESCs media showed the different pluripotent markers, embryo incorporation ability, patterns of gene expression and metabolism features (Fig 6). These characteristics indicated that mpiPSCs resembled naïve state more than hpiPSCs did. These findings may facilitate understanding of the gene regulation network and metabolism of piPSCs and promote the establishment of *bona fide* porcine ES cell lines.

## Supporting Information

S1 Fig Schematic diagram of the reprogramming procedure. (A) Schenatic diagram of the reprogramming protocol. (B) The reprohramming efficiency (AP positive numbers/ starting cell numbers) in hpiPSCs and mpiPSCs.
(TIF)

S2 Fig Genes expression in two types of piPSCs by quantitative RT-PCR assay. (A) Endogenous pluripotent genes expression levels in two types of piPSCs. *p < 0.05, **p < 0.01, ***p < 0.001, (mean ± SD, n = 3) (B) Transgenes expression levels of in both piPSCs. (C) The pluripotent genes expression in mpiPSCs and hpiPSCs. *p < 0.05, **p < 0.01, ***p < 0.001, (mean ± SD, n = 3) (D) Relative expression levels of *Stat3*, *Smad1* and LIFR in mpiPSCs and hpiPSCs. *p < 0.05, **p < 0.01, ***p < 0.001, (mean ± SD, n = 3).
(TIF)

S3 Fig Pearson correlation coefficients of whole genomic expression profiles between hpiPSCs, mpiPSCs and fibroblasts. (TIF)

S1 File Differential genes (fold change >2) in piPSCs versus pEFs. (XLSX)

S1 Table Sequence of primers used in this study. (DOCX)
