# Supplementary figures and images for: Pluripotent and Metabolic Features of Two Types of Porcine iPSCs Derived from Defined Mouse and Human ES Cell Culture Conditions
Source: PLoS One. 2015 Apr 20;10(4):e0124562. doi: 10.1371/journal.pone.0124562 (PMC4404361; doi:10.1371/journal.pone.0124562)

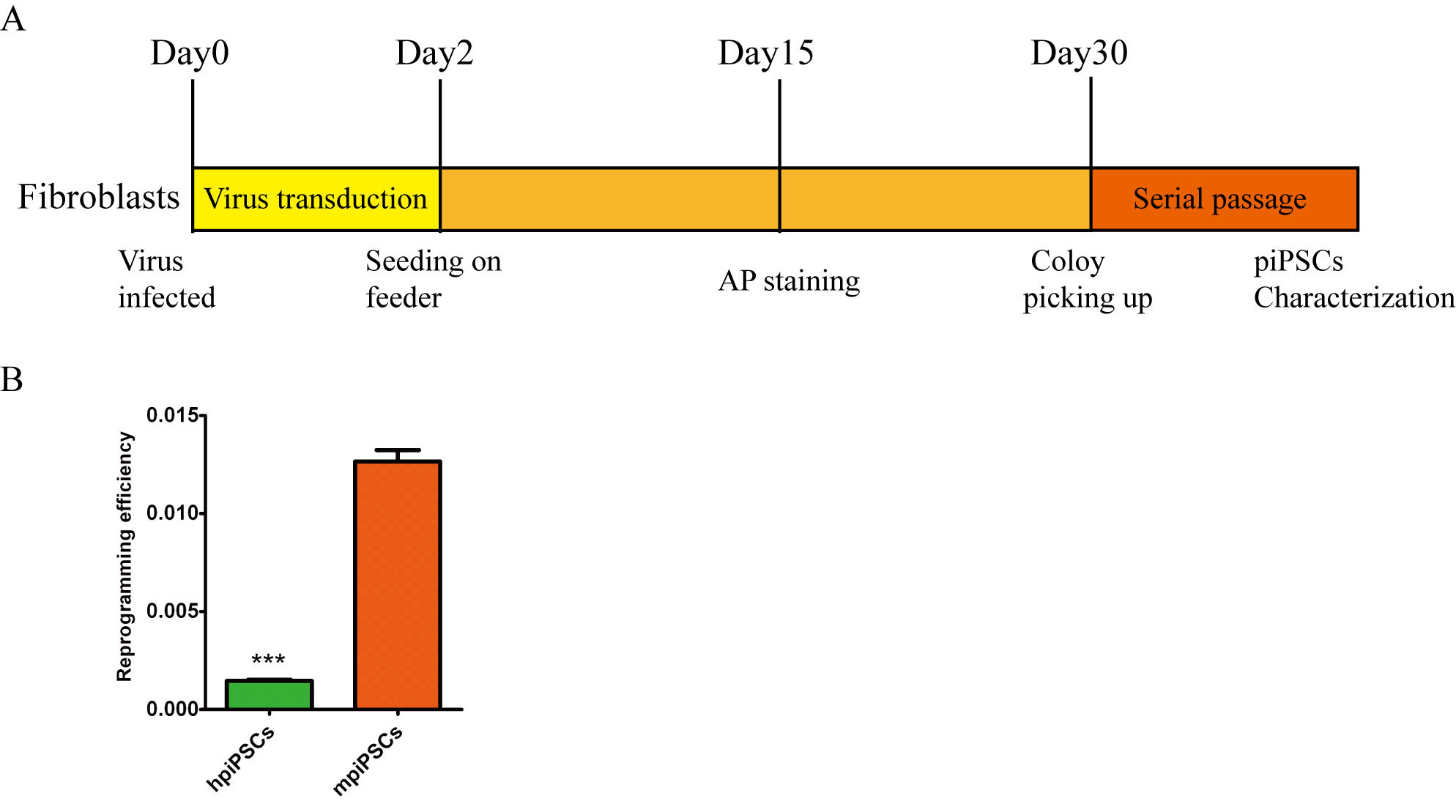

Supplement: S1 Fig — (A) Schenatic diagram of the reprogramming protocol. (B) The reprohramming efficiency (AP positive numbers/ starting cell numbers) in hpiPSCs and mpiPSCs. (TIF) [file pone.0124562.s001.tif]

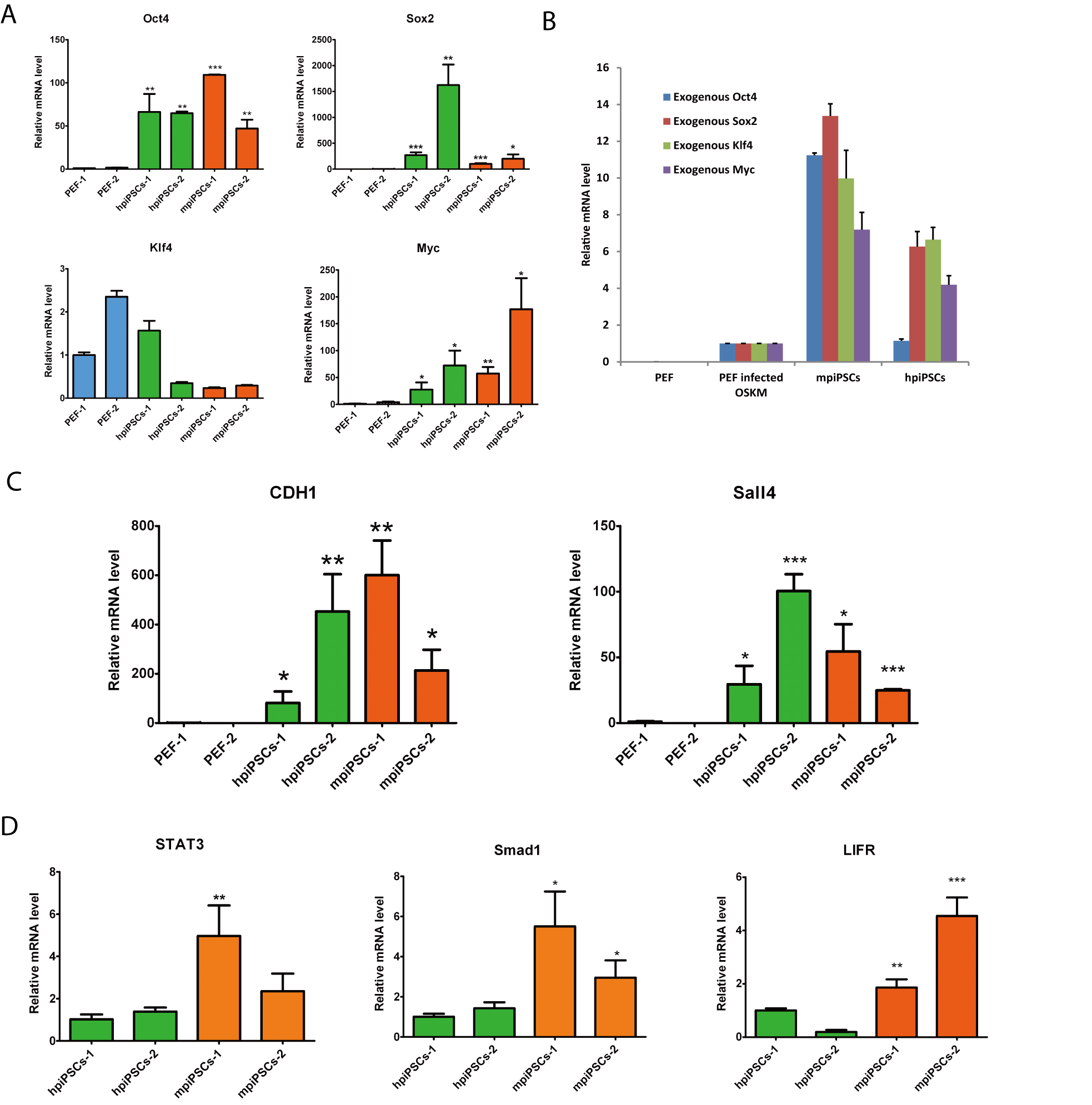

Supplement: S2 Fig — (A) Endogenous pluripotent genes expression levels in two types of piPSCs. *p < 0.05, **p < 0.01, ***p < 0.001, (mean ± SD, n = 3) (B) Transgenes expression levels of in both piPSCs. (C) The pluripotent genes expression in mpiPSCs and hpiPSCs. *p < 0.05, **p < 0.01, ***p < 0.001, (mean ± SD, n = 3) (D) Relative expression levels of Stat3, Smad1 and LIFR in mpiPSCs and hpiPSCs. *p < 0.05, **p < 0.01, ***p < 0.001, (mean ± SD, n = 3). (TIF) [file pone.0124562.s002.tif]

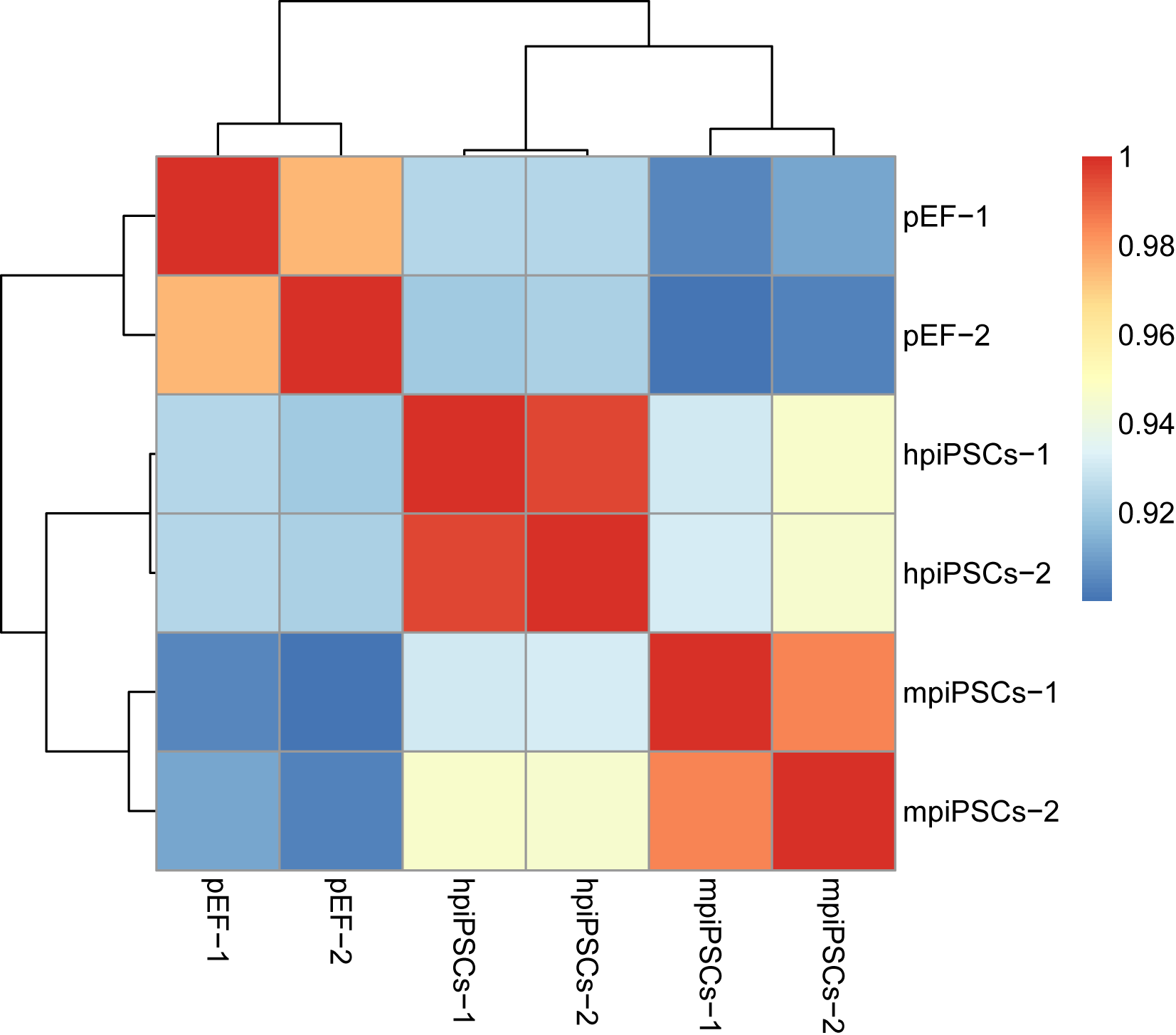

Supplement: S3 Fig — (TIF) [file pone.0124562.s003.tif]
