# Supplementary material for: Pluripotent and Metabolic Features of Two Types of Porcine iPSCs Derived from Defined Mouse and Human ES Cell Culture Conditions
Source: PLoS One. 2015 Apr 20;10(4):e0124562. doi: 10.1371/journal.pone.0124562 (PMC4404361; doi:10.1371/journal.pone.0124562)
Supplement: S1 Table — (DOCX) [file pone.0124562.s005.docx]

| Name | Access No. |  | Sequence (5’-3’) | Size (bp) |
| --- | --- | --- | --- | --- |
| pMXs-Oct4 |  | FW | GACGGCATCGCAGCTTGGATACAC | 120 |
|  |  | RW | GAGAAGGCGAAGTCGGAAG |  |
| pMXs-Sox2 |  | FW | GACGGCATCGCAGCTTGGATACAC | 200 |
|  |  | RW | GGCTGTTCTTCTGGTTGC |  |
| pMXs-Klf4 |  | FW | GACGGCATCGCAGCTTGGATACAC | 200 |
|  |  | RW | GTCTTTGCTTCATGTGGG |  |
| pMXs-Myc |  | FW | GACGGCATCGCAGCTTGGATACAC | 120 |
|  |  | RW | GAAATAAGGCTGCACCGAGT |  |
| Gata6 | NM_214328.2 | FW | CTCCATTCAGACGCCGCTAT | 109 |
|  |  | RW | CTGAGGCCGTTCATCTTGCT |  |
| Nanog | NM_001129971.1 | FW | CATCTGCTGAGACCCTCGAC | 94 |
|  |  | RW | GGGCTTGTGGAAGAATCAGG |  |
| Cpt1b | NM_001007191.1 | FW | AGGCCTCATCAAGAAGTGCC | 183 |
|  |  | RW | TGAACGAAGGCTGTGGACTC |  |
| EF-1α | NM_001097418.1 | FW | AATGCGGTGGGATCGACAAA | 100 |
|  |  | RW | CACGCTCACGTTCAGCCTTT |  |
| Endo-Oct4[[1](#_ENREF_1)] |  | FW | CAAACTGAGGTGCCTGCCCTTC |  |
|  |  | RW | ATTGAACTTCACCTTCCCTCCAACC |  |
| Endo-Sox2[[1](#_ENREF_1)] |  | FW | CATCAACGGTACACTGCCTCTC |  |
|  |  | RW | ACTCTCCTCCCATTTCCCTCTTT |  |
| Endo-Klf4 | NM_001031782.2 | FW | CATGAGTTGGGGGAGGGAAG | 180 |
|  |  | RW | ACTCACCAAGCACCATCGTT |  |
| Endo-Myc[[1](#_ENREF_1)] |  | FW | ATCCAAGACCACCACCACTG |  |
|  |  | RW | GTTCACAGCAACATTCAGGTAGA |  |
| CDH1 | NM_001163060.1 | FW | TGGGCCGAGTGAGTTTTGAA | 82 |
|  |  | RW | TGACTGTAACCACACCGTCG |  |
| Sall4 | NM_001114673.1 | FW | ATCGACGTTTATCCGAGCCC | 165 |
|  |  | RW | TGAGAAGTTCTTCCCGCACC |  |
| Stat3[[2](#_ENREF_2)] | NM_001044580.1 | FW | ATGTGCAGAAAACTCTCACG | 203 |
|  |  | RW | TGGGGTCCCCTTTGTAGCTC |  |
| Smad1[[3](#_ENREF_3)] | NM_213965.1 | FW | GCATCAACCCCTACCACTA | 194 |
|  |  | RW | GAGGAAACGGATGGCTGT |  |
| LIFR[[4](#_ENREF_4)] | SSU91518 | FW | CCCAAATAATGTTGAGGTTCTG | 118 |
|  |  | RW | GTTTTCGGGTTCTGCATCAG |  |

**Supporting information References**

1. Gu Q, Hao J, Hai T, Wang J, Jia Y, et al. (2014) Efficient generation of mouse ESCs-like pig induced pluripotent stem cells. Protein Cell 5: 338-342.

2. Kues WA, Herrmann D, Barg-Kues B, Haridoss S, Nowak-Imialek M, et al. (2013) Derivation and Characterization of Sleeping Beauty Transposon-Mediated Porcine Induced Pluripotent Stem Cells. Stem Cells and Development 22: 124-135.

3. Hall VJ, Hyttel P (2014) Breaking Down Pluripotency in the Porcine Embryo Reveals Both a Premature and Reticent Stem Cell State in the Inner Cell Mass and Unique Expression Profiles of the Naive and Primed Stem Cell States. Stem Cells and Development 23: 2030-2045.

4. Cheng D, Guo Y, Li Z, Liu Y, Gao X, et al. (2012) Porcine induced pluripotent stem cells require LIF and maintain their developmental potential in early stage of embryos. Plos One 7: e51778.
